# Immunosuppression Therapy in Acquired Hemophilia A: Pursuing an Optimal Regimen

**DOI:** 10.7759/cureus.20467

**Published:** 2021-12-16

**Authors:** Dúlio Teixeira Passos, Ana Mafalda Abrantes, Liliana Santos, Ana Cardoso, António Pais de Lacerda

**Affiliations:** 1 Serviço de Medicina II, Centro Hospitalar Universitário Lisboa Norte, Lisbon, PRT; 2 Instituto de Semiótica Clínica, Faculdade de Medicina, Universidade de Lisboa, Lisbon, PRT; 3 Department of Rheumatology, University College London Hospitals, London, GBR; 4 Clínica Universitária de Medicina II, Faculdade de Medicina, Universidade de Lisboa, Lisbon, PRT

**Keywords:** infection, immunosuppressive agents, acquired hemophilia a (aha)

## Abstract

Acquired hemophilia A (AHA) is a rare bleeding disorder occurring mostly in elderly persons, caused by inhibition of factor VIII (FVIII). It is generally detected prior to surgery by an isolated prolonged activated partial thromboplastin time (aPTT) not correcting on mixing studies, with subsequent identification of reduced FVIII levels and presence of FVIII inhibitor. It is treated with hemostatics and immunosuppressants, which may increase the risk for life-threatening opportunistic infections.

A 79-year-old woman with idiopathic acquired FVIII inhibition and severe bleeding presented with anemia, isolated and prolonged aPTT, low FVIII activity (<1%), and elevated FVIII inhibitor titer (471 Bethesda units per milliliter [BU/mL]). Initially, she was treated with recombinant activated factor VII and steroids. However, several hematomas appeared, one of which caused airway compression that required orotracheal intubation. Cyclophosphamide, rituximab (RTX), and activated prothrombin complex concentrate were initiated, resulting in clinical and laboratory resolution after five weeks. Cyclophosphamide and RTX were maintained for six and four weeks more, respectively. After 12 weeks of oral immunosuppression, the patient was readmitted due to antibiotic-resistant *Pseudomonas aeruginosa* sepsis, which resulted in death.

Infection secondary to immunosuppression is the leading cause of death of patients with AHA. In AHA, combination therapy was shown to be more effective than monotherapy, but it was also identified to increase the risk of infection. Age, FVIII activity <1%, and FVIII inhibitor titers >20 BU are predictors of adverse events and poor prognosis in AHA patients. Additional studies are needed to clarify the ideal drug regimens and the need for prophylactic antibiotics in this population.

## Introduction

Acquired hemophilia A (AHA), a rare bleeding disorder caused by autoantibodies against coagulation factor VIII (FVIII), affects 1 in 1,000,000 individuals per year [1]. About half the cases of AHA are considered idiopathic. The risk of AHA is higher in the elderly, during pregnancy, and up to one year after giving birth. The manifestations of the bleeding phenotype of AHA range from mild to life-threatening bleeding. Prior to surgery, acute onset of bleeding with an unexplained, prolonged activated partial thromboplastin time (aPTT) should be investigated. The diagnosis of AHA, including titration of FVIII inhibitor, can be confirmed with a Bethesda assay or enzyme-linked immunosorbent assay with anti-FVIII antibody [2]. Treatment is tailored according to the FVIII inhibitor titer and site and severity of bleeding. For hemostatic treatment, agents such as activated prothrombin complex concentrate (aPCC), recombinant activated FVII (rFVIIa), and human or porcine FVIII are currently the standards [3]. Tranexamic acid can be administered in combination with aPCC and rFVIIa to improve clot stability [4]. Administration of corticosteroids alone or in combination with cyclophosphamide or rituximab (RTX) can induce remission [5].

Here, we report the case of a 79-year-old woman with AHA who succumbed due to sepsis, associated with immunosuppression therapy. This case highlights an evidence-based approach to factor VIII deficiency and management of complications of the disease.

## Case presentation

**Figure 1:**
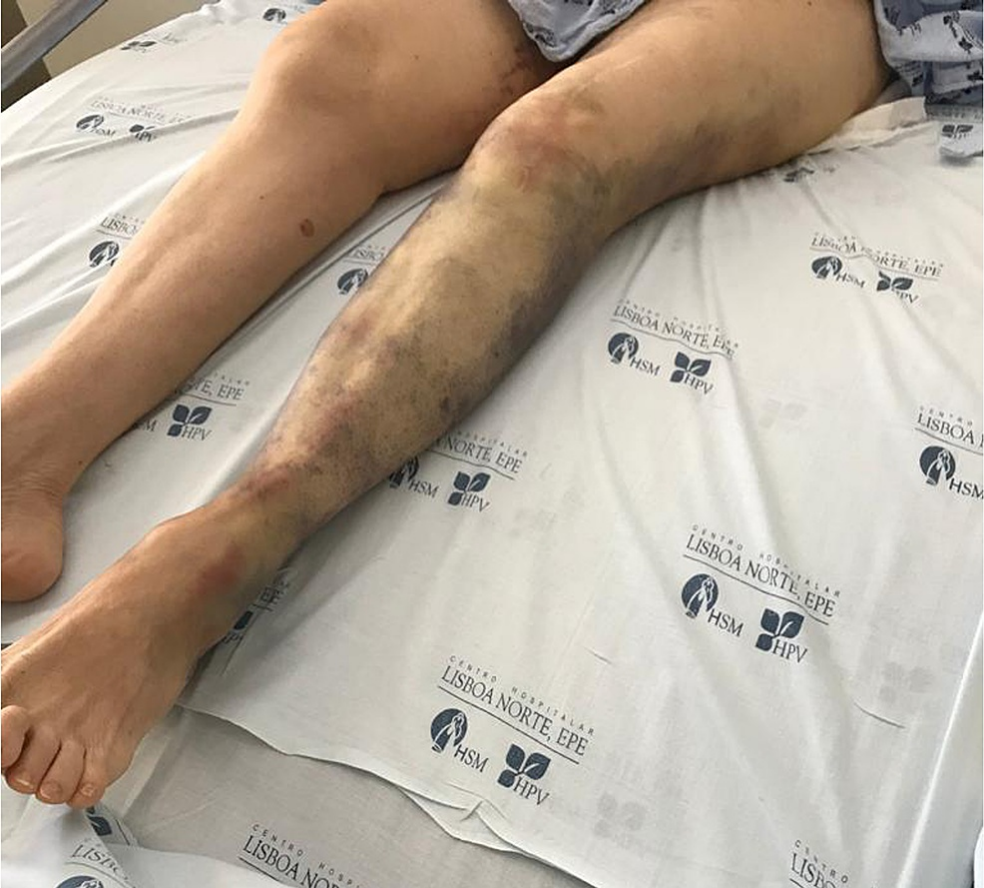
Hematoma of the thigh with extension to the ankle.

**Figure 2:**
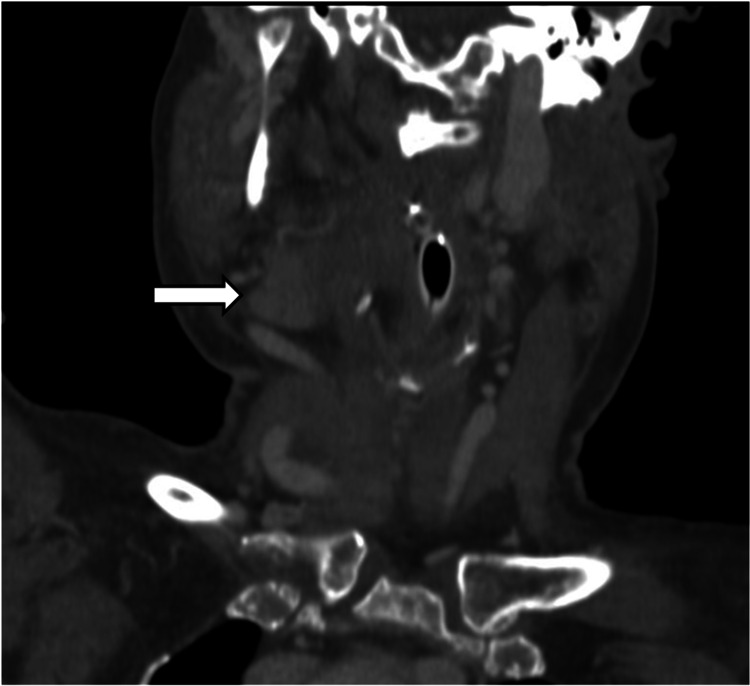
CT showing right cervical hematoma (arrow) with airway compression, coronal view.

A 79-year-old woman presented at our institution with a spontaneous thigh hematoma noticed during the last three days (Figure 1). Three weeks before the presentation, she experienced uncontrolled epistaxis. The patient denied other symptoms, such as hematuria and GI bleeding. Neither did she use anticoagulants/anti-platelets, nor did she have a personal or family history of bleeding or trauma. Laboratory investigations revealed a hemoglobin level of 6.7 g/dL (normal range: 12.0-16.0 g/dL), isolated, prolonged aPTT (>67.3/29 seconds), and a reduced prothrombin time (<8/11.6 seconds). The FVIII activity was low (<1%) and the FVIII inhibitor titer was elevated (471 Bethesda units per milliliter [BU/mL]). Thus, she was diagnosed with AHA and was admitted. HIV, underlying malignancies, or autoimmune disorders were not detected. On the assumption of idiopathic AHA, she was transfused with two units of RBC, rFVIIa (90 µg/kg/dose), and methylprednisolone (1 mg/kg/day), which was switched after five days to prednisolone (60 mg/day). Five days later, she developed a spontaneous hematoma on the right forearm accompanied by mandibular and subconjunctival hematomas. On the sixth day of admission, a brain CT scan and nasofibroscopy revealed right parietal and temporal hematomas and massive pharyngeal hemorrhage (Figure 2). Tranexamic acid was initiated, and anticipating the risk of airway compression, orotracheal intubation was performed. She was then admitted to the ICU. Cyclophosphamide (100 mg/day) and RTX (600 mg/week) were administered along with steroids. Hemostatic control was achieved with aPCC (FEIBA®), and rFVIIa and aPCC were administered for 16 days. After five weeks, the FVIII inhibitor titer was negative and the FVIII titer was 97%, but cyclophosphamide and RTX were maintained for another six and four weeks, respectively. Meanwhile, prednisolone was tapered for 12 weeks, and cotrimoxazole was administered as prophylaxis against infections. Fluconazole was administered for esophageal candidiasis. UTIs caused by *Escherichia coli* and carbapenemase-producing *Klebsiella pneumoniae* were also treated. Two weeks after discharge, the patient was readmitted due to *Pseudomonas aeruginosa* sepsis and shingles. She was refractory to treatment and expired.

## Discussion

AHA is a severe disease that may be fatal, and a patient’s survival depends on early recognition and prompt treatment. Currently, there is no consensus regarding the treatment regimen for AHA [6].

Immunosuppression therapy is a vital component of AHA treatment that allows a shorter time for remission and, therefore, lowers bleeding risk [7]. AHA remission has been defined as a decrease of the FVIII level to within normal range, an undetectable FVIII inhibitor titer, and non-relapse following dose reduction or discontinuation of immunosuppression therapy. Meanwhile, partial remission is assumed when the FVIII level is restored to above 50%, and there is no evidence of bleeding for 24 hours after suspension of hemostatic treatment [8].

Infections represent a significant cause of death in patients with AHA. Most of the infections are caused by immunosuppression therapy [7]. These complications can range from uncomplicated pulmonary or urogenital infections to intra-abdominal sepsis requiring hospitalization, as illustrated in this case [9].

FVIII activity <1% and FVIII inhibitor titer >20 BU, which were noted in this patient, are considered predictors for poor prognosis [7]. According to the international recommendations, patients presenting with these parameters should be treated with corticosteroids combined with a cytotoxic agent or RTX [7]. In the European Acquired Hemophilia Registry (EACH2), RTX and cyclophosphamide combination therapy resulted in a higher percentage of patients achieving complete response [10]. Despite recent studies mentioning the superior efficacy of combined immunosuppressive agents, such as corticosteroids plus cyclophosphamide or RTX, pooled therapy was also identified as a major risk factor for infections [7]. In our case, cyclophosphamide and RTX were administered, as our patient showed life-threatening bleeding and a persistently elevated FVIII inhibitor titer [11]; however, this increased the risk for infection [12].

A recent study has suggested that age is also associated with unfavorable events, highlighting the need for cautious administration of immunosuppressive agents in older patients [12].

This report highlights the complexity of balancing the treatment of a potentially fatal disease with the adverse effects of prolonged immunosuppression, particularly in an elderly patient that must be closely monitored. All opportunistic infections need to be treated promptly and properly. Additional studies are required to clarify the drug-related predictors and the optimal drug regimen for the treatment of AHA and the development of prophylactic antibiotics.

## Conclusions

Infections are known complications related to immunosuppression therapy in AHA. The combination of corticosteroids and cyclophosphamide or RTX has superior efficacy over monotherapy. However, it also significantly increases the risk for infections. Meanwhile, age, FVIII activity <1%, and inhibitor titer >20 BU are considered as predictors for poor prognosis and adverse events in AHA patients. This clinical case clarifies the importance of developing therapies with fewer side effects, especially in older patients, and an urgent need for prophylactic antibiotics.
